# The 60^+^ year journey of ankylosing spondylitis from Rome criteria to today

**DOI:** 10.1515/rir-2026-0025

**Published:** 2026-07-13

**Authors:** Chenching Wu, Wenchan Tsai

**Affiliations:** Department of Internal Medicine, Kaohsiung Medical University Hospital, Taiwan, China

**Keywords:** ankylosing spondylitis, radiographic axial spondyloarthritis, non-radiographic axial spondyloarthritis, sacroiliac joint

## Abstract

It has been more than 60 years since the development of Rome criteria for ankylosing spondylitis (AS). These criteria wonderfully delineated the clinical pictures of AS, and since then, tremendous works has paved the way for the study and management of AS patients. However, their low sensitivity, specificity and the potential for a delay diagnosis remain major problems of these criteria. Over the past six decades, new classification criteria had been developed to address these issues. As is often the case, new solutions bring new challenges. Avoiding over-diagnosis and over-treatment become a crucial contemporary issue. Hopefully, new modifications of classification criteria are on their way to better defining and treating AS patients.

## Introduction

Ankylosing spondylitis (AS) is a chronic inflammatory disease primarily presenting as low back pain. This term has a long and evolving history in medical literature, derived from the Greek words ankylosis (bent or crooked) and spondylos (vertebra). Although the clinical presentations are highly variable-ranging from simple low back pain to complete spinal ankylosis resulting in kyphosis and limited spine movement, this term was widely accepted in the early 20th century.^[1]^

In addition to the clinical presentation, the main diagnostic criteria relied on the presence of radiographic evidence showing the inflammation of sacroiliac joint (SIJ). In most cases, it took more than 10 years to develop radiographic change that meet the criteria for definite radiographic sacroiliitis.^[2,3]^ In the last two decades, Magnetic Resonance Imaging (MRI) has been demonstrated to be capable of detecting early inflammation of SIJ.^[4]^ The Assessment of SpondyloArthritis international Society (ASAS) proposed new classification criteria and recommended changes in the nomenclature pertaining to axial spondyloarthritis (axSpA). Based on image findings, axSpA was further differentiated into radiographic axSpA (r-SpA) and non-radiographic SpA (nr-axSpA).^[5]^ Since then, the terms AS and radiographic axSpA (r-axSpA) have often been used interchangeably. However, an article published in 2024 suggested abandoning the term “AS” for more precisely defining the disease and proposing research programs.^[6]^ It has been more than 60 years since the first official definition of the criteria and term of AS. We will trace back the major milestones in the evolution of these terminology.

## History of AS

AS is an ancient disease, described by more than 20 eponyms throughout history: among them, Bechterew’s disease, Marie-Strumpell’s disease, Morbus Bechterew-Marie-Strumpell, pelvospondylitis, and spondylitis deformans were more frequently used before 20th century.^[7]^ As early as in Greece, Hippocrates (460 BC–370 BC) described a patient with spinal inflexibility and pain.^[8]^ It is interesting to note that the high prevalence of “spondylitis deformans in Egyptian, Roman, and Coptic times.^[9,10]^ although these descriptions could not reliably be determined to be AS due to the lack of solid evidence such as X ray findings or pathologic specimens.

Bernard Connor (1666–1698) is believed to be the first to have clear pathological description of the disease. He detailed the calcification of spinal ligaments, resulting in a fixed, fused spine. He also showed the ankyloses of one knee joint.^[11]^ Two skeletons, dated to about 1500 B. C., were reported 60 years ago to show definite radiological spinal changes of AS, proving the disease was prevalent in ancient Egyptian times.^[12, 13, 14]^ Interestingly, recent report illustrated that among the pharaohs of the 18th and 19th dynasty of Old Egypt, at least three had AS.^[15]^

AS has long been considered as a variant of rheumatoid arthritis (RA) because they shared similar peripheral joint swelling, synovial pathology and elevated erythrocyte sedimentation rate.^[16, 17, 18]^ Opinions against this classification argued that AS chiefly presented with low back pain, many of patients lacked peripheral arthritis, there was a strong familiar tendency, and none of these cases had subcutaneous nodules.^[19, 20, 21]^ The argument settled down after the discovery and widespread application of rheumatoid factor for the diagnosis of RA.^[22,23]^ Furthermore, the advent of X-ray imaging in the early 20th century allowed physicians to correlate clinical symptoms with radiographic changes, focusing on the importance of sacroiliitis and bamboo spine.^[24,25]^ Over subsequent decades, diagnostic criteria evolved. Initial criteria aimed merely to distinguish AS from RA.

### The Classification Criteria of AS

A consensus symposium was held in Rome in 1961 during an EULAR meeting where the first version of the diagnostic criteria of AS, known as “Rome criteria”, was established.^[26]^ It combined the clinical features of chronic inflammatory back pain with radiographic evidence of sacroiliitis. These criteria were far from perfect due to the lack of a solid definition for the limitation of chest expansion (which is age - dependent) and the inclusion of acute anterior uveitis (which had low sensitivity). More importantly, although the radiographic criterion was given a greater weight than the clinical criteria, a diagnosis of AS was still possible based solely on clinical criteria, without any radiographic evidence. However, the concept of inflammatory low back pain was precisely indicated by the presence of low back pain and stiffness for more than 3 months, which could not be relieved by rest.

The New York criteria were introduced in 1966, making modifications of the Rome criteria.^[27]^ In the New York criteria, it was mandatory to have a radiographic criterion +/- clinical criteria. The presence of clinical criteria along with the radiographic one yielded a diagnosis of definite AS; while the absence of clinical criteria led to a diagnosis of probable AS. The New York criteria emphasized the indispensable image finding and required bilateral grades 2 or more or unilateral sacroiliitic lesion grade more than 3 to meet the radiographic requirement. Other modifications included the quantification of the limitation in chest expansion (less than 2.5 cm) and the omission of acute anterior uveitis. Unfortunately, the importance of inflammatory nature of low back pain was not emphasized in this version of classification criteria.

A clinical history screen test with very high sensitivity and specificity was widely used in the late-70 to survey the clinical parameters and attempt an early diagnosis of AS patients^[28]^ It found that the symptom of low back pain must be chronic (longer than 3 months as included in Rome criteria) with relief upon exercise but no subsidence with rest. These findings led to the modification of the New York criteria in 1984, which were more inclusive.^[29,30]^ Table 1 lists the comparison of difference among the three criteria for AS.

**Table 1:** The comparison of difference among 3 criteria for AS

Feature	Rome criteria	New York criteria	Modified New York criteria
Back pain and stiffness	>3 months, not relieved by rest	Limitation of L- spine motion	>3 months, not relieved by rest Improve by exercise Limitation of L- spine motion
Thoracic involvement	required	History of thoracolumbar pain	
Chest expansion	decreased	Limited to 1 inch or less	Decreased relative normal values for age and sex
Radiographic sacroiliitis	Bilateral > 2	Bilateral > 2	Bilateral > 2
	Unilateral > 3	Unilateral > 3	Unilateral > 3
Extraskeletal feature	Uveitis included	Uveitis not included	Uveitis not included
Diagnostic rule	Any clinical plus sacroiliitis	> 1 clinical feature plus sacroiliitis	> 1 clinical feature plus sacroiliitis

AS, ankylosing spondylitis.

### The Discovery of the Association of HLA-B27 with AS

It has been known for years that susceptibility to AS has a familial tendency. Several famous families in history were found to have AS in different generations. Other than the family members of pharaohs mentioned previously, at least four generations of the family members of the Medicis, the most powerful and influential dynasties in European history, especially during the Renaissance were found to be suffered from AS.^[8]^

Two reports published almost simultaneously in 1973 uncovered a strong association between HLA-B27 and AS.^[31,32]^ These findings not only explained the familial tendency of this disease but also led to a tremendous amount of clinical observations and basic researches tried to verify the role of HLA-B27 in the pathogenesis of AS.

The structure of HLA B27 is unique and different from other HLA class I molecules in that one of its 6 antigen–binding pockets, pocket B, has a specific conformation and a nearly absolute restriction for peptide ligands with arginine at position (P) 2.^[33,34]^ HLA-B27-negative AS patients in Japan often carried B39 which has a B pocket conformation similar to that of B27.^[35]^ Furthermore, the transgenic animal model illustrating the major role of B27 molecule in the pathogenesis of inflammatory arthritis^[36]^ pushed the B27 molecular research boom to the climax.^[37]^ These studies illustrated that the interaction of HLA-B27 with environmental factors, such as the microbiome, could be the triggering factor for the disease.^[38, 39, 40]^ Several hypotheses, including molecular mimicry, arthitogenic peptides, modulation of intracellular bacterial survival, and misfolding protein response, had been proposed to clarify the role of HLA-B27 in AS pathogenesis.^[41, 42, 43, 44, 45]^ More recently, focus has been placed on the enzymes responsible for trimming peptides in the endoplasmic reticulum (ER) (ERAP1, ERAP2), the participation of the specific TCRs and tissue phosphatase in the modification of disease spectrum.^[46, 47, 48]^

On the other hand, the clinical observational studies further untangled the role of B27 in the mystery of familial tendency in ankylosing spondylitis. The chances of an HLA B27 positive individual developing AS in a lifetime are quite low (1%–2%). However, the risk increases to 20% for an individual with a first-degree relative (FDR) with AS.^[49]^ A long observational study that followed AS patients with their FDR for 35 years also illustrated that the lifetime relative risk of axSpA for HLA-B27 (+) FDR is 27.1%. 18.2% of children of AS probands were affected,^[50,51]^ and this percentage is higher than that of children of non-radiographic axSpA probands. Of interest, the frequency of AS varies with geographical prevalence of HLA-B27. In some ethnic group, extraordinary high HLA-B27 prevalence was reported to be associated with high frequency of AS.^[52,53]^ Early reports illustrated that some HLA-B27 subtypes carried different risk in different ethnic groups. Among these reports, HLA-B27:04 had a strong association of AS in Chinese populations, and HLA-B27:06 and HLA-B27:09 were considered to have protective effects.^[54, 55, 56]^ Thanks to the use of next-generation sequencing technology, the HLA nomenclature has been updated rapidly, and as of June 2022, the 260 known alleles are numbered HLA-B*27:01 to HLA-B*27:260.^[57]^ Furthermore, the more powerful 3^rd^ generation sequencing method was recently launched, which can generate long reads directly from a single molecule and reveal information about how individual cells function. Using this new technology, we can probably answer more questions about the roles of HLA-B27 in the pathogenesis of AS and its co-morbidities.^[58]^ Better understanding the role of B27 could be crucial for better treatment.^[59]^

## The Concept of Spondyloarthropathy

### The Consolidation of the Concept

In the 1960s, interest in the interrelation between different so-called “variant rheumatoid arthritis” erupted. One of the pioneer reports illustrated the link between Reiter’s syndrome and psoriatic arthritis.^[60]^ The similarity of certain forms of psoriasis and keratodermia blennorrhagica, both clinically and histologically, was well-known in that time. In this report, a high incidence of sacroiilitis and ocular lesion was found in both groups of patients. Before that, growing evidence revealed that the association between psoriasis and arthritis was one of significance rather than coincidence. From the mid-1950s onwards the concept that psoriatic arthritis was a distinct entity was well established.^[61, 62, 63]^ When compared with RA, high incidence of SIJ lesions and even full-blown AS was reported.^[64]^

In the meantime, the American Rheumatism Association took the first step to propose a new classification system in which RA, AS, psoriatic arthritis (PsA), and Reiter’s disease were classified as separate entities.^[65]^ Following this stream, the concept of the association of ulcerative colitis, Crohn’s disease and psoriasis with AS spread like wildfire throughout the entire rheumatology field.^[66, 67, 68]^

Further consolidation of the concept that these diseases were linked together as a special group was built by a series of controlled family studies focusing on inflammatory bowel diseases, PsA and AS. Particularly, they also investigated the prevalence of sacroiilitis in patients and their relatives.^[67, 69, 70, 71]^ Interestingly, overlap of these diseases might happen in a single patient and either disease might precede the development of the other. Even more, overlap of these diseases happened in the relatives of the proband in the pedigree studies. Family members might suffer from different diseases of this special disease group. These studies further confirmed that the prevalence of the linked disease is often higher than can be accounted for by chance, making it reasonable to assume that genetic predisposition plus some environmental stress leads to the development of one or more of these diseases. All these studies were conducted in pre-HLA typing era. Certainly, the discovery of HLA allotypes associated with diseases supports the results of these findings.

Initially, this group of diseases was given the term “sero-negative spondarthritides”. Certain criteria were listed as essential such as absence of ANA, RF and rheumatoid nodules; Inflammatory peripheral arthritis (often asymmetrical), and radiological sacroiliitis, with or without AS. Comorbidities such as skin lesions, oral or genital ulcer. genitourinary infection, and ocular lesions were also included.^[72]^ Other than the diseases mentioned before, Behcet’s disease, Juvenile chronic arthritis and acute anterior uveitis were also listed as member of this disease group. After two decades of collaborative works, a consensus was reached to change the term “spondarthritis” to spondyloarthritide or spondyloarthropathy (SpA).^[73,74]^

### The ESSG and Amor Criteria

An urge to modify the previous criteria for SpA was apparent due to too much restriction of the present criteria. Much heterogeneity among members of SpA was noted in both clinical and X ray features. Sacroliilitis is a mandatory requirement for AS but not an obligate finding in other SpA. On the other hand, some patients presented with typical enthesisits, dactylitis but lacked X ray evidence of SIJ lesion hindering rheumatologists from making a correct diagnosis. Some of these patients were even B27 positive. The term “B27-associated syndrome” or “undifferentiated SpA” had been proposed and discussed in academic circles. The spectrum of SpA was considered wider than the sum of all disease entities mentioned previously.^[75]^

A new preliminary classification criteria of SpA patients, aiming to encompass patients with undifferentiated SpA was proposed in 1991 by The European Spondylarthropathy Study Group (ESSG).^[76]^ Of note, the Amor criteria for SpA was published one year before ESSG criteria.^[77]^ These two sets of criteria have been proposed to discriminate SpA from other rheumatic diseases. However, there were some basic differences between them (Table 2). Basically, Amor criteria were based on scoring system in which different clinical, radiographic features, HLA-B27 status, and drug response contributed different points. A score of 6 or more was considered to fulfill the SpA criteria. In particular, sacroiliitis is one of these criteria having highest score (3 points) and is considered very specific for SpA. However, it is no longer mandatory for this classification system. In ESSG criteria, inflammatory back pain and/or synovitis required as entry criteria. Patients with at least one entry criterion and one minor criterion are classified as having SpA. Surprisingly, uveitis was abandoned in ESSG criteria. The aim of ESSG criteria is not to make a correct diagnosis but to have a wider spectrum of SpA phenotypes, including undifferentiated SpA which was not been proposed in Amor criteria.

**Table 2:** The difference between The ESSG and Amor criteria

ESSG	Amor criteria	
Inflammatory spinal pain or any one of the following	Inflammatory back pain	1 point
scroiliitis	Unilateral buttock pain	1 point
Alternative buttock pain	Alternative buttock pain	2 point
Enthesitis	enthesitis	2 point
	Peripheral arthritis	2 point
	dactylitis	2 point
IBD	Acute anterior uveitis	2 point
Positive family history of spondyloarthropathy	HLA-B27 positive or family history of spondyloarthropathy	2 point
	Good response to NSAID	2 point

Diagnosis of spondyloarthropathy with 6 or more points. ESSG: European spondyloarthropathy group; IBD: inflammatory bowel diseases; ESSG: The European Spondylarthropathy Study Group.

Both criteria have been evaluated in many ethnicities and localities. Their performance was quite similar and with a high degree of sensitivity and specificity. The sensitivity and specificity for ESSG is 83.0%–98.0% and 87.0%–96.0% respectively.^[78, 79, 80, 81]^ For the Amor criteria, the sensitivity was 88.5%–90.8%, specificity was 91.9%–96.2%.^[82,83]^ However, in some studies, the performance of the ESSG criteria was moderate: only 46.6% of patients with possible SpA who all met the ESSG criteria at entry into the study were judged to have SpA after 5 years of follow up.^[84]^ The difference among various investigation reports may suggest the impact of environmental stress and heterogeneous genetic back ground.

From Rome criteria to ESSG criteria, radiographic sacroiliitis is a hallmark features of all these proposals. However, there are two important problems: (1) X ray cannot show active inflammation, the result of X ray finding is structural damage not an inflammatory status, though we can still say that the structural damage is the result of previous or present continuous inflammation. (2) It takes years to have structural damage after inflammation. By the time that the X ray finding becomes visible, patients may already have symptoms for years without a definite diagnosis and treatment. In particular, the finding in AS of male predominance and more peripheral joint involvement in female was refuted to be due to lower detection rate of SI joint damage in females. This could be attributed to the masking effect of the female pelvic organ or sacroiliitis with low structural damage. Hence, the cry for new imaging technology and modification of criteria appealed one after another.^[85]^

## The Evolution of axSpA

### The Use of Magnetic Resonance Imaging (MRI)

In the early 1990 s, the use of MRI to detect sacroiliac joint lesion sprung up like mushrooms.^[86, 87, 88, 89, 90]^ More than 50% of sacroiliitis can be demonstrated by MRI in SpA patients in whom abnormalities are not revealed by conventional radiography.^[91]^ However, the specificity and predictive value of MRI lesions for the axial SpA were soon challenged. The SIJ is a complex and unique joint composed of an anterior/inferior cartilaginous compartment and a posterior/superior ligamentous compartment. Compared to other weight bearing joints which are horizontal in plane (like intervertebral joints, knee joint), SIJ is the only vertical weight bearing joint of our body. Its primary function is to transmit the load between axial skeleton and lower limbs. Hence, in athletes, laborers with heavy work, considerable strain may put on the SIJ, which may lead to early mechanical and degenerative changes.^[92]^ On the other hand, the stress of pregnancy may also lead considerable inflammation in this joint.^[93]^ Other conditions such as infection, and osteitis condensa also contribute to some MRI image change of SIJ.^[94, 95, 96]^

To solve these concerns, the assessment of spondyloarthritis international society (ASAS) /Outcomes Measures in Rheumatology (OMERACT) working group published a consensus definition for ‘active sacroiliitis by MRI’ in 2009.^[97]^ There are two components in this this definition, a qualitative component (bone marrow edema [BME] highly suggestive of axSpA) and a quantitative component (one BME lesion on two consecutive MRI slices or more than one BME on a single slice). However, insufficient specificity of the quantitative component of the 2009 MRI cut-offs was raised. 20%–40% of healthy individuals and those with non-specific back disorders were reported to have false-positive BME lesions meeting the 2009 definition.^[98, 99, 100]^ A new MRI definition was proposed in 2021, Using the “data-driven” method they derived new cutoffs that were either > 4 SI joint quadrants with BME at any location or at the same location in > 3 consecutive slices. The other component, structural lesions, were defined as: (1) to have any one of > 3 SI joint quadrants with erosion, or (2) > 5 with fat lesions, erosion at the same location for > 2 consecutive slices, fat lesions at the same location for > 3 consecutive slices, or (3) presence of a deep (*i.e*. > 1 cm depth) fat lesion.^[101]^ Both the short-tau inversion recovery (STIR) and contrast-enhanced fat-suppressed T1 (for the depiction of the inflammatory lesions) and the T1W (for the structural lesions) sequences were considered for qualitative evaluation of the images. These MRI lesion cut-offs had very high (95%) PPV. Follow up study found that the new definition might increase the specificity of the quantitative part of the 2009 MRI inflammatory lesion definition.^[102]^

### The ASAS Criteria for Axial Spondyloarthritis

It has long been known that many of AS patients might had symptom years before the appearance of radiographic change of the SIJ. Some proposals and terms had been published trying to reduce the diagnostic delay and to give these patients earlier and appropriate treatment.^[103,104]^ This call became more demanding after the approval of biologic agents for treating AS patients. Tumor necrosis factor inhibitors were demonstrated to be powerful and effective medications to reduce spinal inflammation and hence the symptoms and signs of AS. These medications were optimistically considered not only to reduce patients’ suffering from pain and burdens but also to prevent the radiographic progression and the development of full-blown AS.

**Figure 1:**
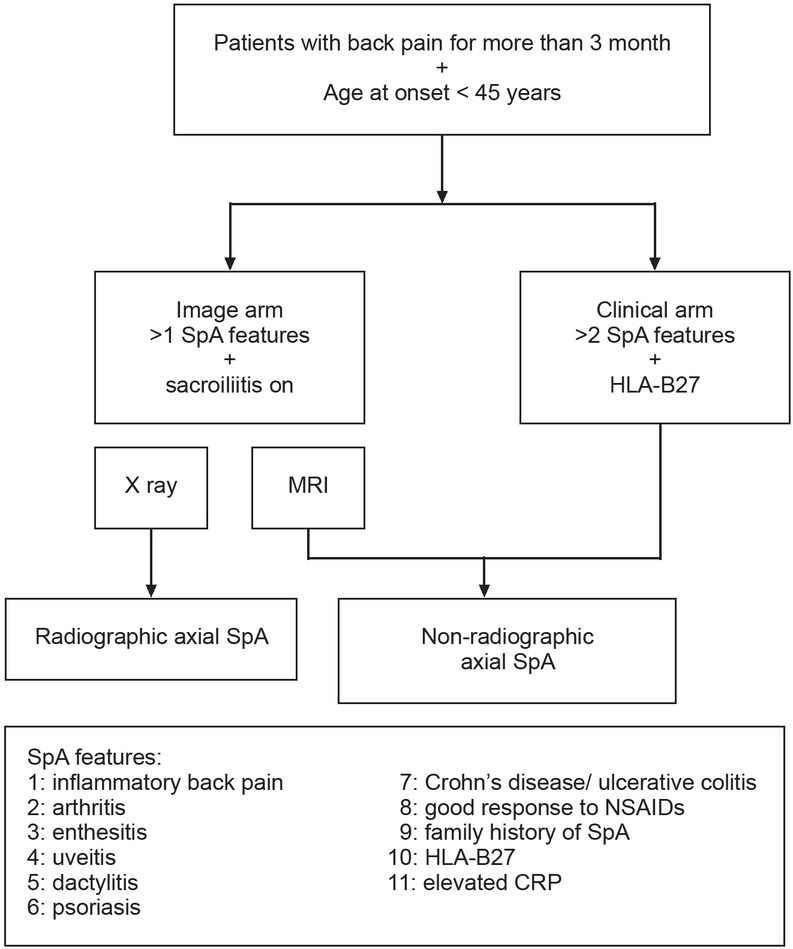
The ASAS criteria for axial spondyloarthritis. Modified from original ASAS classification criteria. ASAS: Assessment of spondyloarthritis international society; SpA: spondyloarthritis; MRI: magnetic resonance imaging; NSAIDs, nonsteroidal anti-inflammatory drugs; HLA-B27, human leukocyte antige-B27; CRP, C-reactive protein.

To incorporate full spectrum of patients (AS and Pre-AS), the Assessment of Spondyloarthritis International Society (ASAS) developed a new classification criteria and adapted the term: axial spondyloarthritis (axSpA) to encompass all patients^[105,106]^ (See Figure 1). Under the umbrella of axSpA, there are two major arms: image arm and clinical arm. To be classified as SpA, patients must have at least one SpA features if they have positive image finding (either X ray or MRI), or they must have at least 2 SpA features plus positive HLA-B27 typing. In addition, based on whether the radiographic change of SIJ existed or not, patients were sub-grouped into radiographic ax-SpA (r-axSpA) and non-radiographic axSpA (nr-axSpA). Among these, r-axSpA was proposed as an alternative diagnostic term for AS.

These criteria were shown to have high sensitivity (82%) and specificity (89%).^[107,108]^ However, these multi-arm design classification criteria were soon criticized for having decreased the homogeneity (which was the most important quality of a classification criteria) of the patient population. The sensitivity and specificity were 66.2 and 97.3 % respectively for the imaging arm alone. The corresponding figures for the clinical arm were 56.6 and 83.3 %, respectively. Other cohort observations also revealed the difference between two arms.^[109]^

Questions raised about the position of nr-axSpA, do those patients represent early cases of AS? Several studies have explored possible differences between nr-axSpA and r-axSpA in both clinical and demographic aspects.^[110]^ Theoretically, the age of symptom onset in nr-axSpA should occur earlier than r-axSpA, however in a Swiss cohort r-axSpA had an earlier age of symptom onset.^[111]^ The previous report was not alone, a meta-analysis finding followed this result (27.8 y/o *vs*. 26.3 y/o for nr-axSpA and r-axSpA respectively).^[112]^ Sex ratio and prevalence of HLA-B27 positivity are also different between these two groups.^[113, 114, 115, 116]^ However, a report also showed that both diseases shared the same burden but differed in the prevalence of peripheral joint involvement.^[117]^

For those nr-axSpA patients who were considered could be a case of AS in the future, analyses from GESPIC, have shown that only a small percentage (12%) of them progressed to AS in 2 years. Increased level of CRP was found to be a risk factor.^[118]^ Other cohorts had shown different incidence rate of about 10%–40%.^[119]^ However, several observations also support the important role of family history in the speed of radiographic progression.^[120,121]^

Another important question that whether the radiographic progress could be reduced by the advanced therapy in nr-axSpA patients? A PREVENT study showed that at week 104, only 3.3% and 2.9% of patients in the secukinumab and placebo-secukinumab groups, respectively, were re-scored as mNY-positive. Overall, 1.7% and 3.4% of patients developed new syndesmophytes in the secukinumab and placebo-secukinumab group, respectively over 2 years.^[122]^ In the RAPID-axSpA study, patients treated with certolizumab: after 4 years, 4.5% of patients became to fulfill the mNY criteria.^[123]^ A 10-year follow-up study to evaluate sacroiliac radiographic progression of early axSpA showed that the net % progression (from nr-axSpA to r-axSpA) was 5.8%. The probability of meeting mNY criteria for AS was estimated to increase by 0.87% per year. For those received TNF inhibitors the probability was 0.45%.^[124]^

Soon after the development of ASAS criteria for nr-axSpA, clinical trials of several tumor necrosis factor (TNF) inbitors including adalimumab, etanercept and golimumab^[125, 126, 127]^ were conducted in these patients. These TNF inhibitors were approved in EMA for the indication of nr-axSpA. However, these indications were rejected by US FDA. The FDA raised concerns about the inconsistency of radiographic reading of the SIJ and the use of these medication in patients without strong evidence of inflammation (some of these patients in the so-called clinical arm had normal CRP levels).^[128]^ Years later, the US FDA approved the indication of certolizumab pego,^[129]^ two IL-17 inhibitors, secukinumab and ixekizumab^[130,131]^ and one Janus kinase inhibitor (upadacitinib)^[132]^ for nr-axSpA patient with evidence of inflammation (either positive MRI finding or abnormal CRP level).

## Conclusion

It has been more than 60 years since the first official definition of the criteria and term for AS. Despite huge success in the pathogenesis, epidemiology, genetics, and treatment strategy in the past six decades, we still have long way to go to settle several questions that remained to be solved. One of them is to make an early and accurate diagnosis of AS. The ASAS criteria was supposed to achieve early diagnosis, but unfortunately, the risk of over-diagnosis and over-treatment needs to be confronted. Avoiding over-treatment is an important issue in Asian-Pacific countries, because the medication is expensive and risk of deterioration of existing infectious diseases such as tuberculosis, hepatitis should be addressed. Additionally, the viewpoint titled “Good bye to the term ‘ankylosing spondylitis’”, should be reconsidered. According to several cohort studies, more than 90% of patients are mutually inclusive for both AS and r-axSpA criteria. Since the terms AS and r-axSpA are interchangeable in the past two decades, it is unreasonable to abandon “AS”, an old and familial term rather than using r-axSpA. Not to mention the reported prevalence of axial disease in psoriatic arthritis ranged about 25%–70%.^[133,134]^ Some of them could completely fulfill the r-axSpA criteria. It is quite clear that two diseases are different. We are now at the crossroad of managing AS. We have a handful of weapons to combat the old disease, but we still have to avoid over- treatment.
